# Combined analysis of whole‐exon sequencing and lncRNA sequencing in type 2 diabetes mellitus patients with obesity

**DOI:** 10.1111/jcmm.14932

**Published:** 2020-01-19

**Authors:** Tian An, Jing Zhang, Yu‐Fei Liu, Yan‐Xiang Wu, Juan Lian, Ting‐Ye Wang, Yuan‐yuan Hu, Jia‐jian Zhu, Jiangpinghao Huang, Dan‐Dan Zhao, Fang‐Fang Mo, Si‐Hua Gao, Guang‐Jian Jiang

**Affiliations:** ^1^ Traditional Chinese Medicine School Beijing University of Chinese Medicine Beijing China; ^2^ Department of Endocrinology Tangshan People's Hospital Tangshan China; ^3^ The Third Affiliated Hospital Beijing University of Chinese Medicine Beijing China; ^4^ University of Southern California Los Angeles CA USA

**Keywords:** diabetes, long non‐coding RNA, mutation sites, obesity

## Abstract

This study sought to find more exon mutation sites and lncRNA candidates associated with type 2 diabetes mellitus (T2DM) patients with obesity (O‐T2DM). We used O‐T2DM patients and healthy individuals to detect mutations in their peripheral blood by whole‐exon sequencing. And changes in lncRNA expression caused by mutation sites were studied at the RNA level. Then, we performed GO analysis and KEGG pathway analysis. We found a total of 277 377 mutation sites between O‐T2DM and healthy individuals. Then, we performed a DNA‐RNA joint analysis. Based on the screening of harmful sites, 30 mutant genes shared in O‐T2DM patients were screened. At the RNA level, mutations of 106 differentially expressed genes were displayed. Finally, a consensus mutation site and differential expression consensus gene screening were performed. In the current study, the results revealed significant differences in exon sites in peripheral blood between O‐T2DM and healthy individuals, which may play an important role in the pathogenesis of O‐T2DM by affecting the expression of the corresponding lncRNA. This study provides clues to the molecular mechanisms of metabolic disorders in O‐T2DM patients at the DNA and RNA levels, as well as biomarkers of the risk of these disorders.

**Bullet points**

What is already known about this subject? Please remember to also include this between the title page and structure abstract in your paper.

1. Analysis of mutations in the whole‐exon site in peripheral blood of patients with O‐T2DM
2. Screening for common mutant expression genes in O‐T2DM patients
3. A series of metabolically sensitive lncRNA and mutation sites were defined in the peripheral blood of patients with O‐T2DM using whole‐exon sequencing and lncRNA sequencing.

What does this study add? Please remember to also include between the title page and structured abstract in your paper.

1. Mapping of related mutation sites and pathways in patients with O‐T2DM
2. We constructed a PPI co‐expression network in O‐T2DM patients
3. Screen for candidate proteins and sites as potential biomarkers for O‐T2DM patients.

## 1 INTRODUCTION

Whole‐genome exon sequencing technology (WES) is a genomic analysis method that uses target sequence capture technology to capture DNA from all exon regions of the genome for high‐throughput sequencing and has a high sensitivity for identifying disease‐related low‐frequency and rare mutations.^1^, ^2^ WES usually through three major steps finds the pathogenic genes, including exosome capture enrichment, high‐throughput sequencing and bioinformatic data analysis.^3^ In recent years, there have been a number of studies using whole‐genome exon sequencing to screen type 2 diabetes mellitus (T2DM) susceptibility genes. Albrechtsen^4^ sequenced the WES of 2000 Danish populations in three stages and found that the microtubule‐actin crosslinking factor 1 (MACF1) 2290 amino acid was replaced by methionine to proline (M2290V) will increase the risk of T2DM.

T2DM is a chronic endocrine metabolic disease characterized by disorders of carbohydrate, fat and protein metabolism, which pathogenesis is closely related to environmental and genetic factors.^5^, ^6^ With the development of modern social economy and lifestyle changes, the incidence of diabetes is getting higher and higher. T2DM occurs mostly between 35 and 40 years old, accounting for more than 90% of diabetic patients and affecting more than 400 million people worldwide.^7^ Obesity type 2 diabetes mellitus (O‐T2DM) usually refers to T2DM with a body mass index (BMI) that meets the criteria for overweight or obesity. Obesity is the main independent risk factor for T2DM, accounting for 80%‐90% of the causes of diabetes.^8^ Obesity enhances insulin resistance and causes hyperinsulinemia in patients with T2DM. Therefore, the treatment of O‐T2DM is relatively difficult. Individual risk of O‐T2DM is strongly influenced by genetic factors.^9^, ^10^ As a complex metabolic disease, O‐T2DM has a distinct family history, so it is of great significance to explore its pathogenesis from the perspective of genetics.

Therefore, this study analysed the specific DNA mutation sites between O‐T2DM patients and healthy people, and integrated DNA and RNA analyses, combined with functional enrichment and metabolic pathway analysis to explore its pathogenesis, in order to provide references for the diagnosis and treatment of T2DM patients and reveal the biological basis of O‐T2DM.

## 2 MATERIALS AND METHODS

### 2.1 Sample information description

This study was approved by the Ethics Committee of Beijing University of Chinese Medicine (BUCM) and Beijing Hepingli Hospital. All procedures are carried out in accordance with the Helsinki Declaration. All participants received written informed consent. Selection criteria of O‐T2DM participants according to the American Diabetes Association criteria (ADA, 2018). Briefly, fasting plasma glucose (FPG) ≥7.0 mmol/L, glycated haemoglobin (HbA1C) ≥6.5% and body mass index (BMI) ≥25 kg/m^2^. Participants were excluded if they had a history of gestational diabetes, type 1 diabetes; acute primary complications of the heart, liver, kidney, lungs, brain and other organs; and acute complications such as diabetic ketosis and infection. Participants were recruited by Beijing Hepingli Hospital from December 2017 to May 2018. All enrolled participants were divided into O‐T2DM group (Group 1; patient ID: LSr001, LSr002, LSr003, LSr004, LSr005 and LSr006) and healthy crowd (HC) (Group 2; patient ID: LZC001, LZC002, LZC003, LZC004, LZC005 and LZC006). After the subjects were enrolled, venous blood was collected from both groups on a fasting day for subsequent experiments.

### 2.2 DNA extraction and sequencing

DNA samples were evaluated by agarose gel electrophoresis and Qubit analysis. This study used Agilent's liquid‐phase chip capture system to efficiently enrich human all exon region DNA and then perform high‐throughput sequencing on the Illumina platform. The library and capture experiments were performed using the Agilent SureSelect Human All Exon V6 kit, following the instructions and the latest optimized protocol.

### 2.3 Bioinformatics analysis

After obtaining the sequenced reads, the bioinformatics analysis was carried out in the presence of GRCh37 or hg19. It generally includes the following parts: sequencing data quality assessment and mutation detection.

#### 2.3.1 Screening of mutation site

The single nucleotide polymorphisms (SNP)/InDel (insertion and deletion) detected by the basic analysis was subjected to mutation site screening. Firstly, filter the 1000 human genome (1000G) database and retain the mutation site with frequency less than 0.01 in 1000G. Secondly, the variation of the exonic or the splicing region is retained. Thirdly, remove synonymous mutations (mutations that do not result in altered amino acid coding), leaving mutations that have an effect on the gene expression product. Finally, the mutation sites were screened according to the scoring predictions of the four softwares: SIFT, Polyphen, MutationTaster and CADD. Requiring at least half of the four softwares to support this site may be harmful and the site is retained.

#### 2.3.2 Mutation site harmful classification

According to the standards and guidelines for sequence variation proposed by the American Society of Medical Genetics and Genomics (ACMG),^11^ the mutations are classified into five types: pathogenic, likely pathogenic, uncertain significance, like benign and benign.

#### 2.3.3 Copy number variations (CNV) analysis

Similar to single nucleotide variants (SNVs), many CNVs are normal polymorphisms in the biological genome, and this benign CNV does not cause pathological changes in the organism. However, some malignant CNVs have also been found to be associated with diseases such as nervous system disorders and cancer. In order to filter out benign CNV from the CNV results detected by the software, we use the DGV and CNVD databases to classify the detection results.

### 2.4 DNA and RNA conjoint analyses

In order to screen out the true diabetes‐related mutations from the massive variation test results, we need to further analyse and screen the mutation detection results. The most significant GO entries and pathways involved in the mutated gene were determined by significant enrichment analysis. In addition, we used a precise algorithm, combined with sequencing results and a variety of databases to screen and sort candidate genes to construct a correlation map between gene‐DHS ‐diabetes. Finally, the online software GeneMania was used to perform protein functional interaction network analysis of candidate diabetes‐related mutant genes, including protein‐protein and protein‐DNA‐genetic interactions.

### 2.5 Statistical analysis

The statistical differences were analysed using the SPSS (version 20.0, IBM SPSS Statistics) by independent‐samples *t* test. All data were shown as the means ± SEM. *P* values < .05 were regarded as statistically significant.

## 3 RESULTS

### 3.1 Participants information description

Our study enrolled 6 O‐T2DM patients and 6 healthy crowds. All O‐T2DM patients met the diagnostic criteria of fasting plasma glucose (FPG) ≥7.0 mmol/L and glycated haemoglobin (HbA1C) ≥6.5%, as well as the BMI ≥ 25 kg/m^2^. In our study, the mean BMI differed between the two groups significantly, with an average of 25.92 kg/m^2^ in the O‐T2DM patients and an average of 22.99 kg/m^2^ in the healthy subjects (Table 1).

**Table 1:** Clinical characteristics of the participants

Characteristics	DM‐DHSS patients	Healthy crowd	*P*‐value
Age(y)	47.0 ± 4.5	43.5 ± 6.0	.281
BMI (kg/m^2^)	25.92 ± 2.73	22.99 ± 1.39	.041*

Note

n = 6, values are presented as mean ± SD. Significant differences by *P < .05

Abbreviation: BMI, body mass index.

### 3.2 Whole‐exome sequencing data summary

Twelve samples of fasting whole blood were collected: WSR001‐WSR006 for DM‐DHSS patients and WZC001‐WZC006 for healthy crowd. WES showed that a total of 158.36 Gb raw data were obtained. In these raw data, the average error rate was 0.1% and the Q30 content was more than 87.91% (Table S1). The valid WES data were aligned to the reference genome (GRCh37/hg19) by BWA and sort the results using SAMtools comparison. Then, use Picard mark duplicate reads. Finally, in the current study of 12 samples, the mapped content and the fraction of target covered with at least 10X content were more than 99.54% and more than 99.2%, respectively. The average sequencing depth of the target region in the 12 samples of our study was 138.35 (Table S2).

### 3.3 Variation detection results

Based on the mapped results, we used SAMtools to identify single nucleotide variant (SNV) sites and filter SNV sites. A total of 277 377 SNV sites were found in exonic. Among these SNV sites, 100 SNV sites belong to the stoploss type, which means that the substitution codon of the base becomes a non‐stop codon due to substitution by one base. Subsequently, we used ANNOVAR software to annotate the SNP, which covers the location information, type and conservative prediction of the mutation. Insertion and deletion (InDel) occurring at the coding region or splice site may alter the translation of the protein. Therefore, we separately counted the number of different types of InDel on the genome and coding region (Tables S3 and S4).

### 3.4 Screening of mutation sites and classification of their harmfulness

In addition, we performed mutation site screening on the SNP/InDel information detected by the basic analysis and finally obtained 5607 mutation sites. Based on the priority level of the disease, here we list the top ten mutation sites (Table 2). We refer to ACMG's evidence to classify the harmfulness of the mutation sites. The number of mutation sites for each of the harmful categories obtained from the bioinformatics analysis is shown in Table 3. Finally, we performed a structural variation hazard analysis and a total of 160 sites were found (Appendix S1).

**Table 2:** Top ten mutation sites

Priority	POS	avsnp147	GeneName	ExonicFunc	Gencode	KEGG PATHWAY
H	955 677	rs757604648	AGRN	missense SNV	ENST00000379370.2	KEGG ECM RECEPTOR INTERACTION
H	976 598	rs200607541	AGRN	missense SNV	ENST00000379370.2	KEGG ECM RECEPTOR INTERACTION
H	979 560	rs762554040	AGRN	missense SNV	ENST00000379370.2	KEGG ECM RECEPTOR INTERACTION
H	1 221 564	rs61740392	SCNN1D	missense SNV	ENST00000379116.5 ENST00000325425.8 ENST00000400928.3 ENST00000338555.2	.
H	1 233 779	rs544359869	ACAP3	missense SNV	ENST00000353662.3 ENST00000354700.5	KEGG ENDOCYTOSIS
H	1 262 682	rs766592849	CPTP	missense SNV	ENST00000343938.4 ENST00000464957.1	.
H	1 262 875	rs564546199	CPTP	missense SNV	ENST00000343938.4 ENST00000464957.1	.
H	1 269 024	.	TAS1R3	missense SNV	ENST00000339381.5	KEGG TASTE TRANSDUCTION
H	1 269 399	rs571862161	TAS1R3	missense SNV	ENST00000339381.5	KEGG TASTE TRANSDUCTION
H	1 269 623	rs199779671	TAS1R3	missense SNV	ENST00000339381.5	KEGG TASTE TRANSDUCTION

**Table 3:** Harmful classification screening results

Total	Pathogenic	Likely Pathogenic	VUS	Likely Benign	Benign
27 029	22	10	2670	0	24 327

### 3.5 Mutant gene screening shared between samples

On the basis of filtering the harmful parts, the common mutation sites between the two groups were screened according to the principle that 10% of the patients shared and 90% of the control groups did not share. A total of 454 mutation sites were screened in O‐T2DM patients compared to healthy controls. As shown in Table 4, we list the top ten consensus mutant genes.

**Table 4:** The top 10 shared mutant genes and annotation results

Priority	CHROM	POS	avsnp147	QUAL	GeneName	ExonicFunc
H	1	11 848 391	rs763465988	228	C1orf167	missense SNV
H	2	54 482 702	.	228	TSPYL6	non‐frameshift deletion
H	2	54 482 716	rs751318047	228	TSPYL6	non‐frameshift deletion
H	5	64 747 447	rs147540204	228	ADAMTS6	missense SNV
H	5	82 815 317	rs186214606	228	VCAN	missense SNV
H	5	156 479 553	rs773539537	228	HAVCR1	non‐frameshift deletion
H	5	156 479 569	.	228	HAVCR1	non‐frameshift insertion
H	5	156 479 570	.	228	HAVCR1	non‐frameshift insertion
H	9	16 435 821	rs140694690	228	BNC2	missense SNV
H	11	2 436 559	rs80326119	228	TRPM5	missense SNV

### 3.6 Bioinformatics analysis of mutation sites

**Figure 1:**
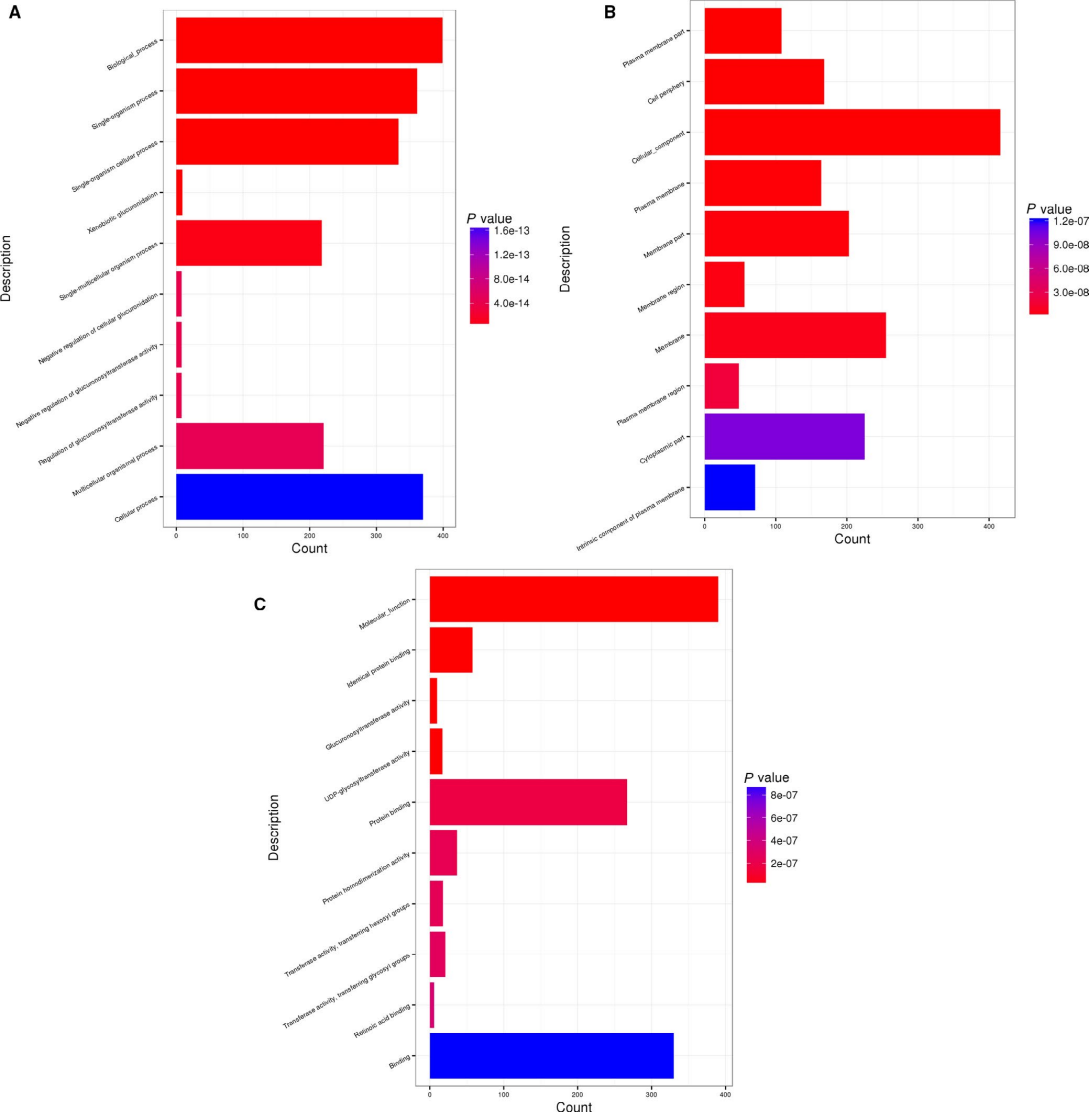
The histogram of GO enriched. A, Biological process, B, cellular component and C, molecular function

Genes perform their biological functions by co‐ordinating each other, especially for the complex disease of T2DM, which may be a phenotypic difference caused by mutation of multiple genes. Therefore, we identified the most important metabolic pathways and signalling pathways involved in mutant genes through significant enrichment analysis. We performed GO enrichment analysis on shared mutant genes from three categories: biological process (BP), cellular component (CC) and molecular function (MF). Based on the *P* value, we list the top 10 entries (Figure 1). The main enriched BP entries are as follows: single‐organism cellular process, single‐multicellular organism process and multicellular organismal process. The main enriched MF entries are as follows: protein binding and protein homodimerization activity; the main enriched CC entries are as follows: cytoplasmic part, membrane part and cell periphery.

**Figure 2:**
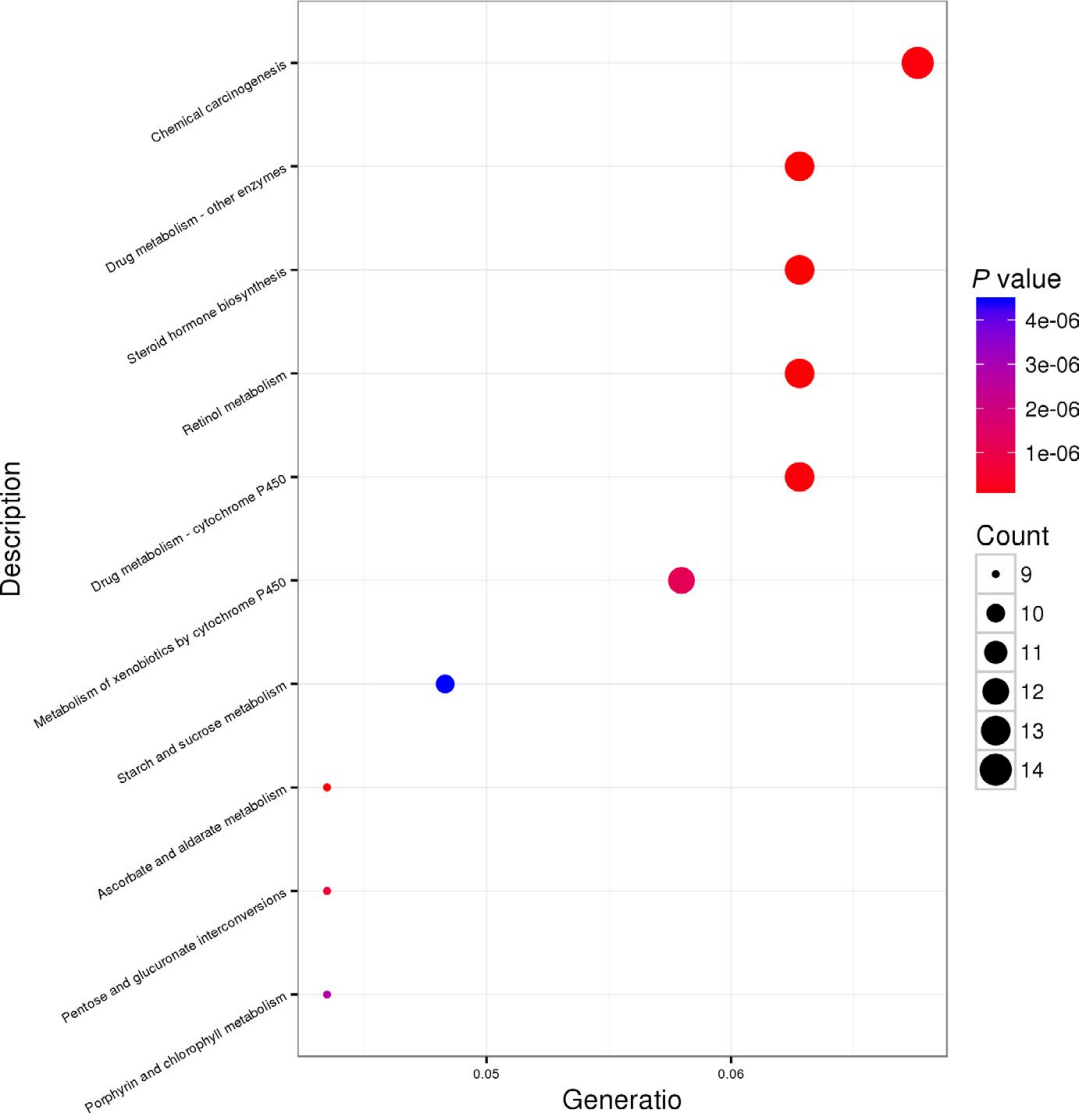
Scatter plot of the KEGG pathway enrichment. Abscissa indicates the proportion of genes enriched in the pathway to the total enriched gene, and the ordinate indicates the name of the enriched KEGG pathway. The dot size indicates the number of genes enriched in the pathway, and the colour indicates the *P*‐value

In addition, we used the KEGG database to perform pathway enrichment analysis of consensus mutations in O‐T2DM patients. The results showed that these mutation sites were significantly enriched in 25 pathways (*P* < .05). Here, we list the top 10 pathways through the scatter plot image (Figure 2). Among them, pentose and glucuronate interconversions, starch and sucrose metabolism are closely related to glucose metabolism.

### 3.7 Gene‐disease phenotype correlation analysis

**Figure 3:**
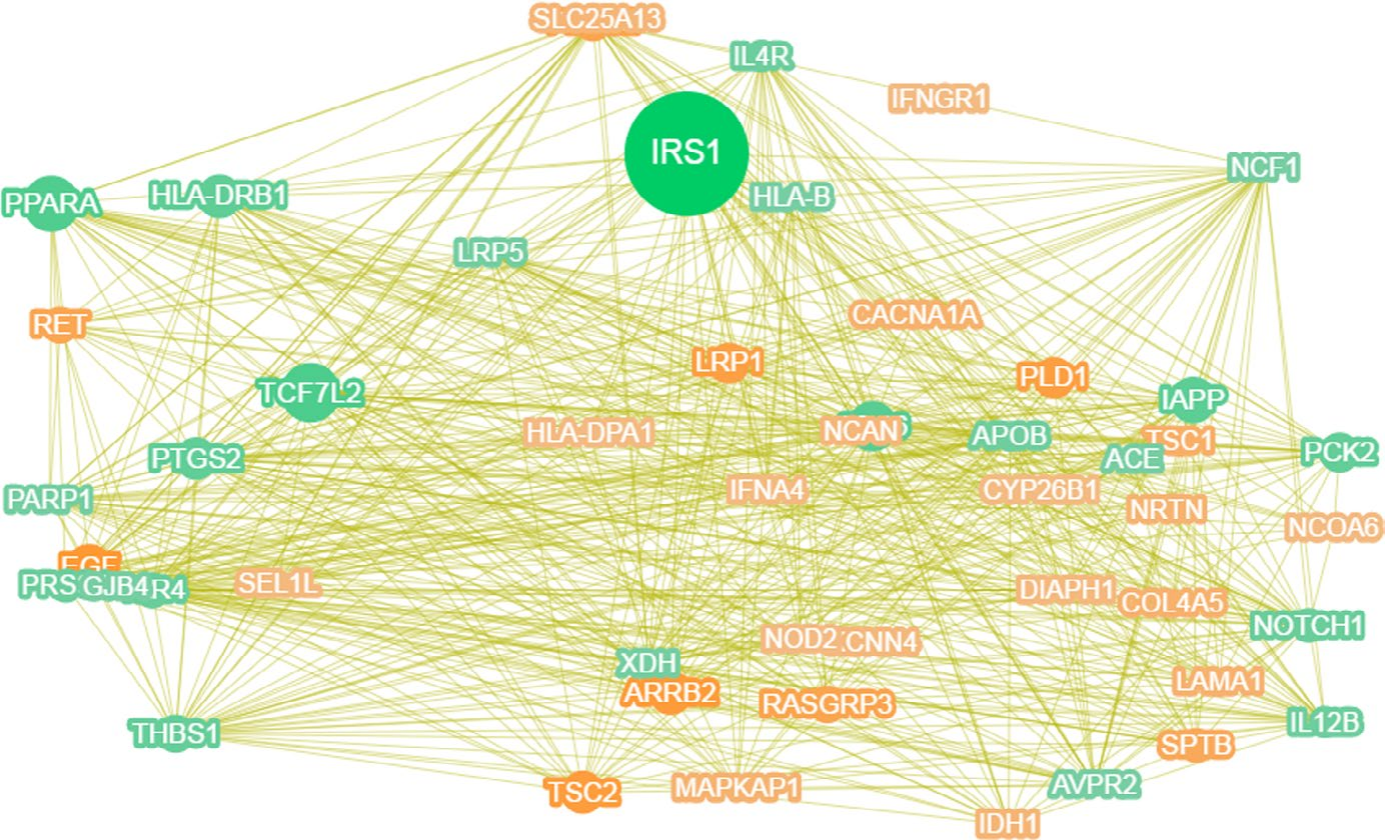
Gene‐phenotype‐disease association network. The size of the shape covered by the gene name represents the strength of the association with the disease, and the larger the shape coverage area, the stronger the correlation with the disease. A Green dot indicates a gene that is considered to be associated with a related disease in an existing report or database; an orange dot indicates a gene that is considered to be related to a green gene based on various associations

**Figure 4:**
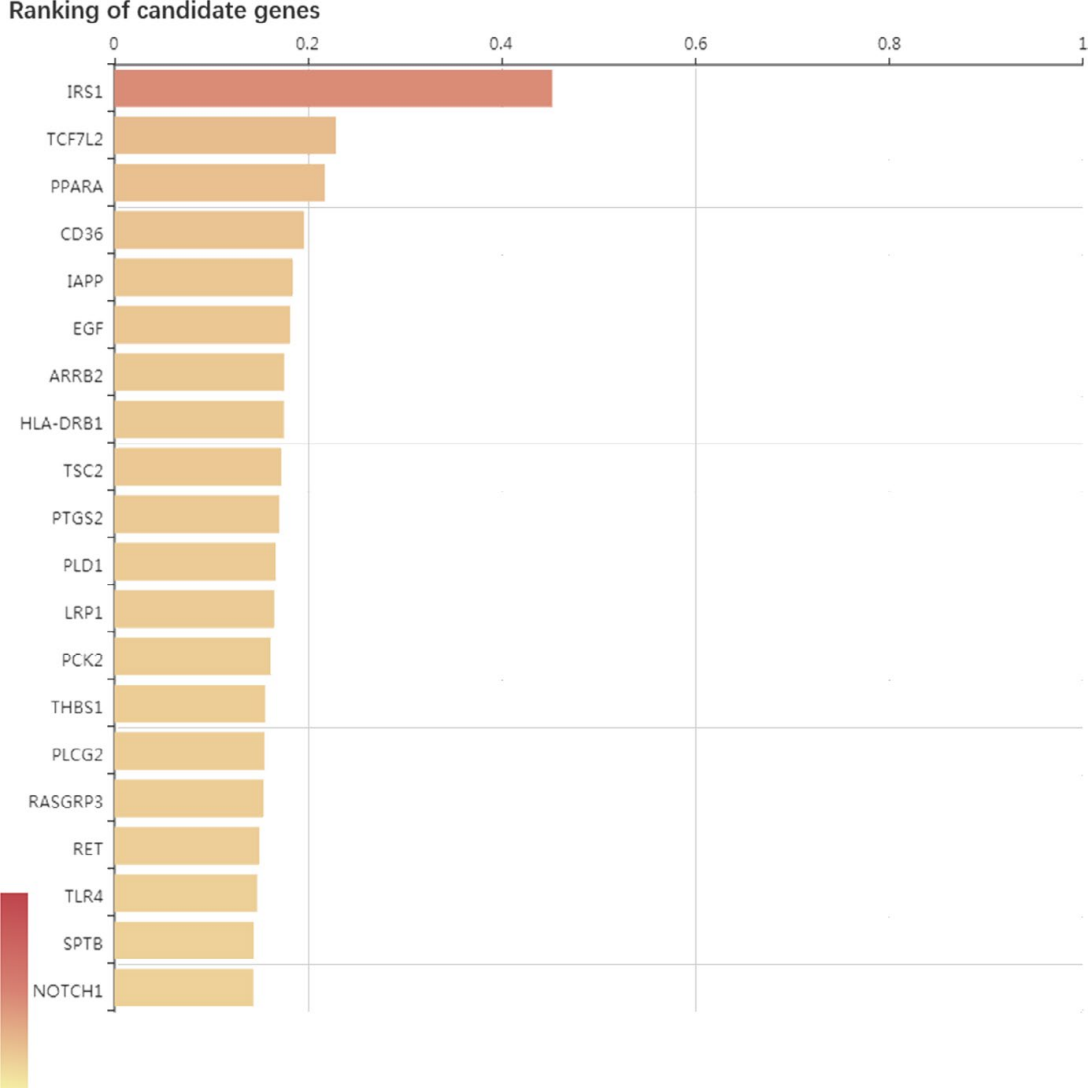
Top 20 candidate genes ranked in association with O‐T2DM. The relevance score is 1 for the maximum

In this study, in order to determine the correlation between candidate genes and diseases, we compared the sequencing results to multiple gene databases (including 561, 119 gene‐disease association records and 135 588 mutation‐disease association records) for correlation analysis. By comparison with the database, a total of 22 472 genes were found to be related to the pathogenesis of T2DM in our sequencing results. Subsequently, we filtered and sorted the candidate genes, screened the first 416 genes and constructed a gene‐phenotype‐O‐T2DM interaction network (Figure 3). In addition, we used Phenolyzer software to rank candidate genes. The higher the ranking, the more likely it is associated with O‐T2DM. Here, we list the top 20 significantly related genes (Figure 4).

### 3.8 Protein function interaction analysis

**Figure 5:**
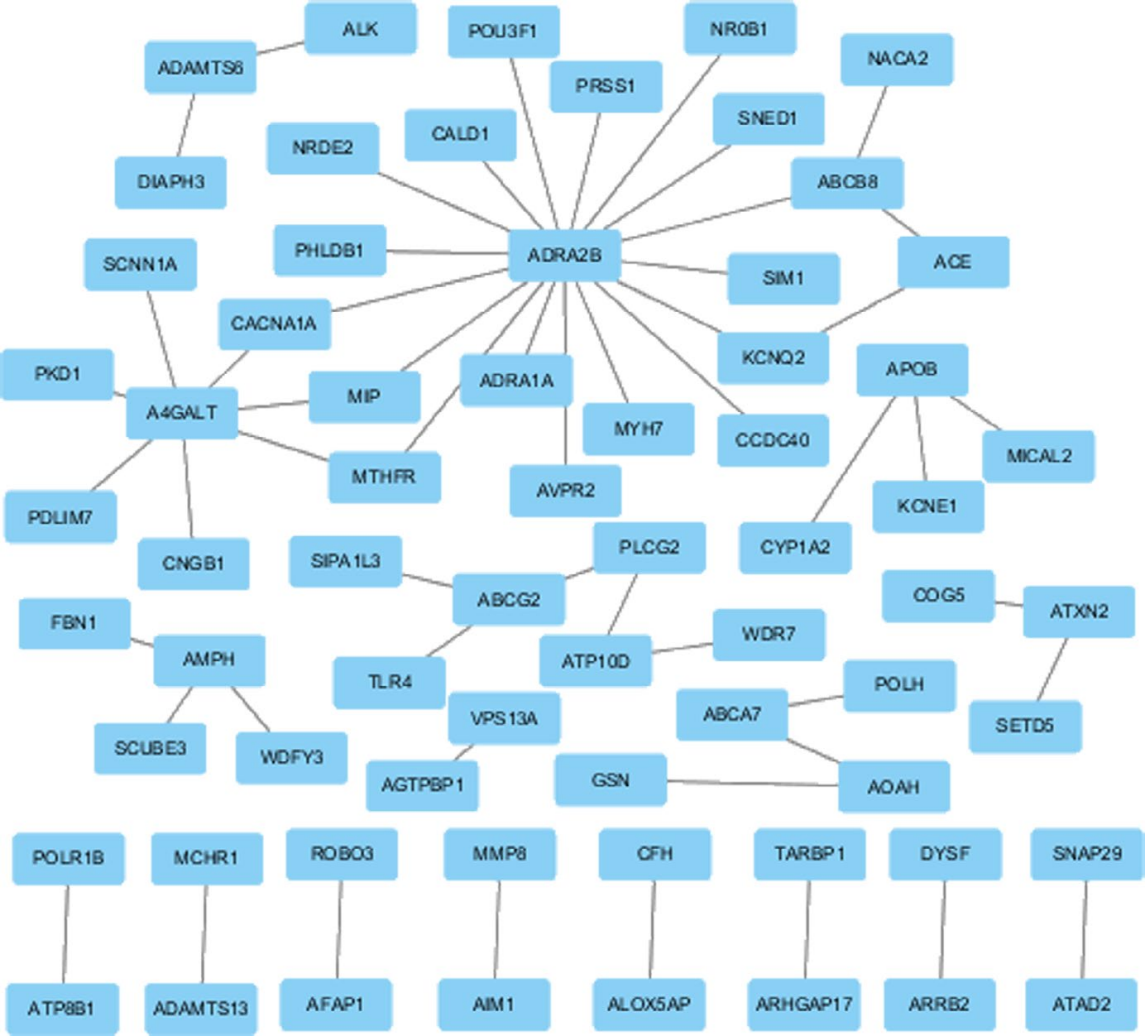
Co‐expression network

We used online software GeneMania^12^ to perform protein functional interaction network analysis of candidate genes, including protein‐protein, protein‐DNA‐genetic interactions, pathways, reactions, gene‐protein expression data, protein domains‐phenotypic screening profiles. Then, use Cytoscape software to construct the co‐expression network (Figure 5). As shown in Figure 5, a total of 21 genes and 54 co‐expressed proteins associated with them are included.

### 3.9 DNA and RNA conjoint analysis results

#### 3.9.1 Mutant gene expression levels shared between samples

**Figure 6:**
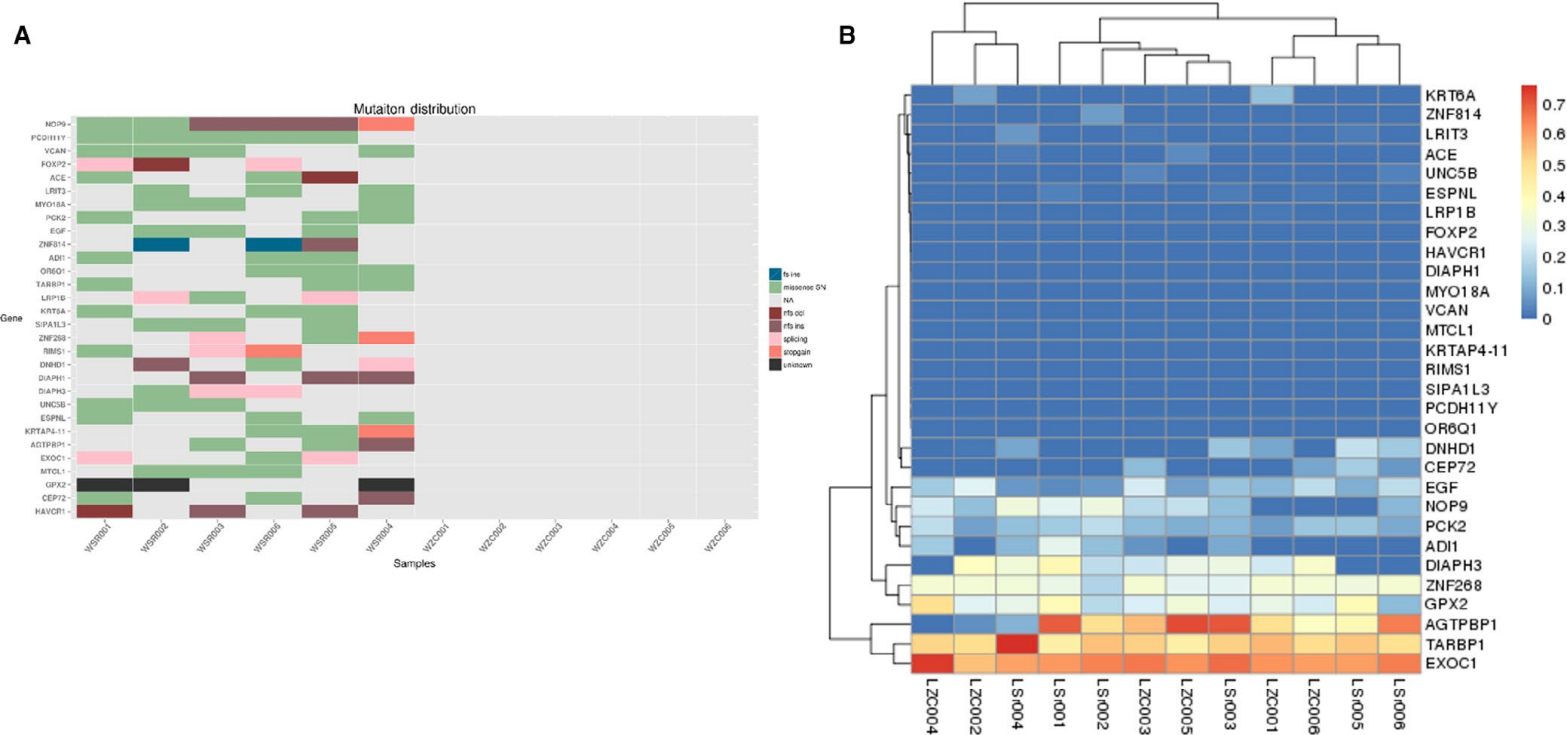
Changes in the expression levels of mutant genes and mutant genes in O‐T2DM patients. A, Mutant genes shared by O‐T2DM patients. The heat map of the consensus mutation gene obtained by screening at the genome level, different colours indicate different mutation types. B, Heat map of mutated gene expression levels in O‐T2DM patients. This figure shows the change in the corresponding expression level of the high frequency mutant gene, expressed as FPKM, and the colour from red to blue indicates the FPKM from large to small

Among all the consensus mutation genes in the O‐T2DM group, NOP9 has mutations in all six patients with O‐T2DM. This was followed by PCDH11Y, which showed mutations in peripheral blood samples from five patients (Table 5). Subsequently, we analysed the expression of these mutant genes at the RNA level. Quantitative analysis of gene levels was performed using cutffdiff software, and the depth of sequencing and gene length were corrected, and the expression values of genes were expressed by FPKM. On this basis, we can visualize the expression level of the transcriptional level of the mutant gene shared by the patient and not in the normal population (Figure 6).

**Table 5:** Consensus mutation genes in the O‐T2DM group

Gene Name	Patient Num	Patient
NOP9	6	WSR001, WSR002, WSR003, WSR004, WSR005, WSR006
PCDH11Y	5	WSR001, WSR002, WSR003, WSR005, WSR006
VCAN	4	WSR001, WSR002, WSR003, WSR004
PCK2	3	WSR001, WSR004, WSR005
EGF	3	WSR002, WSR003, WSR005
LRP1B	3	WSR002, WSR003, WSR005
ZNF268	3	WSR003, WSR004, WSR005
DIAPH1	3	WSR003, WSR004, WSR005
DIAPH3	3	WSR002, WSR003, WSR006
CEP72	3	WSR001, WSR004, WSR006

#### 3.9.2 Screening of common mutant genes and differentially expressed consensus genes in O‐T2DM patients

**Figure 7:**
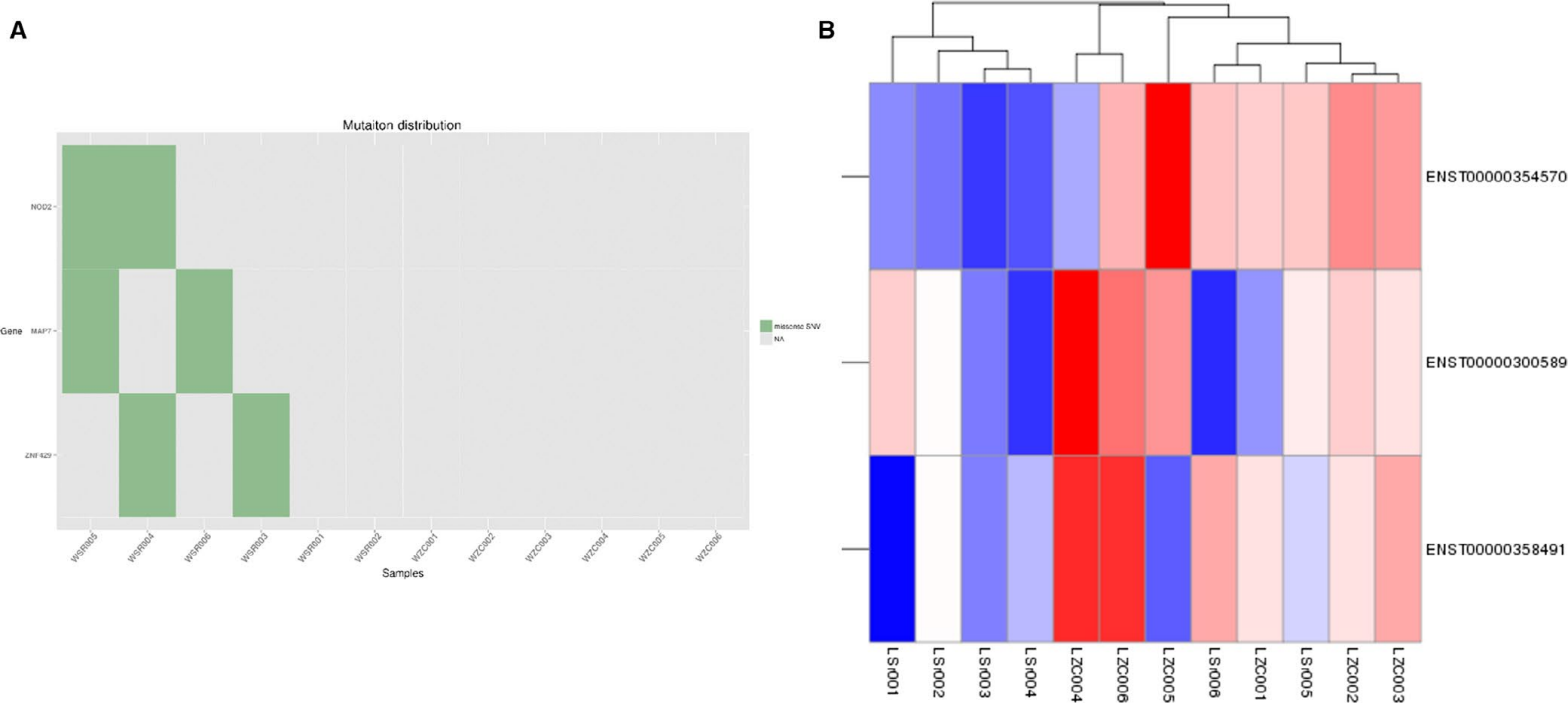
Heat map of mutant genes and differentially expressed genes in O‐T2DM patients. A, Mutant genes. B, Significant differentially expressed genes

If a gene is judged to be a patient‐associated mutant gene at the genome level, and its expression at the RNA level is also significantly different from that of a normal population, the gene may be a functionally important gene. In the current study, we screened 3 genes in O‐T2DM patients for common mutations at the DNA level and differentially expressed genes at the RNA level. They are MAP7, NOD2 and ZNF429, respectively (Table 6, Figure 7).

**Table 6:** Mutant genes and differentially expressed genes in O‐T2DM patients

Consensus mutant gene	Number of patients	Patient number	Corresponding transcript name	*P* value of DE RNA	*q* value of DE RNA
MAP7	2	WSR005, WSR006	ENST00000354570	.01555	0.999325
NOD2	2	WSR004, WSR005	ENST00000300589	5e−05	0.0264352
ZNF429	2	WSR003, WSR004	ENST00000358491	.01795	0.999325

Abbreviation: DE RNA, differentially expressed RNA.

#### 3.9.3 Pathway analysis of mutant genes and differentially expressed genes in O‐T2DM patients

Based on the above analysis, the key genes selected, and all the genes at the genomic level, and the genes differentially expressed at the transcriptional level, were screened for common pathways for enrichment analysis. As shown in Table 7, a total of three pathways are associated with these differentially expressed genes and lncRNAs.

**Table 7:** Pathways of mutant genes and differentially expressed genes in O‐T2DM patients

Pathway ID	Pathway name	DNA name	RNA name
hsa04940	Type I diabetes mellitus	HLA‐B/HLA‐DRB1/HLA‐DPA1/IL12B	FAS|GAD1|GZMB|GZMH|PRF1|FASLG|HLA‐DRB5
hsa05164	Influenza A	IFNGR1/HLA‐DRB1/DDX58/TLR4/PRSS1/STAT2/CASP1/IFNA4/HLA‐DPA1/IL12B/NLRX1	DNAJC3|STAT2|OAS3|OAS2|EIF2AK4|OAS1|EIF2AK2|CCL2|RSAD2|CXCL10|IFIH1|PML|IL6|DDX58|CREBBP|TNFSF10|PIK3CA|ADAR|NFKB1|MAP2K3|IRF7|ACTG1|MX1|KPNA2|NXT2|FAS|PIK3R5|DDX58|OAS3|TNFSF10|IRF7|STAT2|RSAD2|CCL2|CXCL10|IFIH1|ACTG1|PML|OAS2|TNFRSF1A|MX1|CASP1|OAS1|IL6|EIF2AK2|KPNA2|DDX58|EIF2AK2|FASLG|CXCL8|CXCL10|IFIH1|OAS3|SLC25A6|HLA‐DRB5
hsa05330	Allograft rejection	HLA‐B/HLA‐DRB1/HLA‐DPA1/IL12B	FAS|GZMB|GZMH|PRF1|FASLG|HLA‐DRB5

## 4 DISCUSSION

O‐T2DM is a polygenic genetic disease. To date, more than one hundred O‐T2DM susceptibility genes have been obtained from GWAS in the O‐T2DM population. It is currently believed that most of the functional variants are hidden in exons^13^ and are caused by low‐frequency and rare mutations.^14^ However, GWAS is not sensitive to low‐frequency mutations and rare mutations, which may lead to partial information missing. Whole‐genome exon sequencing has a high sensitivity to the discovery of disease‐related low‐frequency and rare mutations.^15^ The low‐frequency mutation means that the minor allele frequency (MIF) is equal to or greater than 0.5% and less than 5%, and the rare mutation means that the allele mutation frequency is less than 0.5%. Since whole‐genome exon sequencing can effectively identify genetic susceptibility genes and mutation sites of complex diseases, it is widely used in the molecular mechanism research and molecular diagnosis of human diseases.

In this study, we performed a full‐exome sequencing of 12 samples (6 normal subjects and 6 obese diabetic patients) using WES technology. And compared with the existing database (dbSNP database, thousand human genome plan, etc), the pathogenicity of the gene mutation site is graded and the mutant gene is screened. These screened genes were then subjected to enrichment analysis and gene‐disease phenotypic correlation analysis. It is hoped that we will clarify the pathogenesis of T2DM and provide gene and molecular targets in clinical diagnosis and diagnosis of T2DM.

We used high‐throughput sequencing to reveal the pathogenesis of O‐T2DM from an exome perspective and look for consensus mutations in O‐T2DM patients. We found a total of 455 mutation sites. Among them, CPTP (ceramide‐1‐phosphate transfer protein) is a member of the GLTP (glycolipid transfer protein) family. Studies have shown that CPTP can act as an endogenous regulator of the production and release of pro‐inflammatory cytokines such as interleukin‐1β and interleukin‐18.^16^ CPTP can induce adhesion molecules to express other cytoplasmic signals through mesenchymal cells and endothelial cells, and induce the infiltration of inflammatory cells and immune cells. In this study, we found that CPTP produced a missense mutation in the exon region. We speculate that the mutation in CPTP may be closely related to the regulation of the immune system in O‐T2DM patients.

Pathway analysis revealed that the consensus mutations are closely related to several important pathways interrelated with O‐T2DM occur, including steroid hormone biosynthesis, starch and sucrose metabolism, pentose and glucuronate interconversions. Steroid hormones, also known as steroid hormones, play an important role in maintaining life function and regulating immunity.^17^ Steroids are a factor in regulating obesity. Steroid hormones can affect the energy metabolism in adipose tissue by regulating the immune tissue population of adipose tissue and increase the total amount of fat.^18^ This study found that the shared mutation gene in O‐T2DM patients is closely related to steroid hormones, which may be related to steroid hormone metabolism disorders. In addition, the pathways of starch and sucrose metabolism, pentose and glucuronic interconversions are important pathways involved in energy metabolism in the human body. Therefore, we speculate that the mutant gene in O‐T2DM patients may affect the occurrence of O‐T2DM disease by affecting the above‐mentioned pathways involved in glycolipid metabolism. In addition, we supplemented the key O‐T2DM signal transduction events in which DNA/RNAs that have undergone important changes in this study are involved in (Appendix S2).

In the study of disease, it is important to determine the association of candidate genes with disease. Combining sequencing results with various databases, we filter and sort candidate genes to construct a correlation map between gene‐phenotype‐O‐T2DM. By comparing with the database, a total of 22 472 genes were found to correlate with the pathogenesis of T2DM in our sequencing results. In addition, we use the Phenolyzer software to rank the candidate genes. The results showed that the top three genes in the association were IRS1 (insulin receptor substrate 1), TCF7L2 (transcription factor 7 analogue 2) and PPARA (peroxisome proliferator‐activated receptor‐α gene).

As an important mediator of insulin binding to its receptors and exerting biological effects, IRS1 plays an important role in the control of blood glucose homeostasis.^19^ A recent study suggests that the cause of insulin deficiency in obese patients may be due to a weakened IRS1 signal.^20^ A study of African‐Americans found that IRS1 mutations and endocrine disorders caused by obesity synergistically reduce insulin sensitivity, suggesting that IRS1 variability and obesity together become an important predictor of insulin resistance.^21^ In this study, we found that IRS1 produced a non‐frameshift insertion mutation in the exonic region of O‐T2DM patients and that IRS1 mRNA expression levels were up‐regulated in O‐T2DM patients compared with healthy controls (Log2fold‐change = 0.11). Therefore, based on the close relationship between IRS1 gene and obesity and lipid metabolism, we believe that IRS1 may be a promising target for clinical prediction of O‐T2DM.

Transcription factor 7‐like 2 (TCF7L2) gene is closely related to T2DM and obesity.^22^, ^23^, ^24^ Patients with a single nucleotide polymorphism (SNP) TT or TC genotype in the TCF7L2 gene are 2 to 1.4 times more likely to have T2DM than CC homozygous patients.^25^ Studies have shown that TCF7L2 can play a significant role in regulating adipose tissue and pancreas via the WNT signalling pathway.^26^ A recent study found that overweight and TCF7L2 were also significantly associated with T2DM.^27^ In our current study, we found missense mutations in the TCF7L2 rs138649767 locus in O‐T2DM patients, and the expression of TCF7L2 was also significantly down‐regulated in the mRNA expression level (log2fold‐change = −99.12). Therefore, we hypothesized that changes in the expression of TCF7L2 post‐transcriptional levels caused by mutations in the TFc7L2 exon region may be closely related to the pathogenesis of O‐T2DM. Peroxisome proliferator‐activated receptor‐α (PPAR‐α) is a class of transcription factors in the PPAR family. PPAR regulates several biological processes in obesity, including inflammation, lipid metabolism and glucose metabolism.^28^ In the regulation of lipid metabolism, it mainly plays a role in affecting lipogenesis, lipid storage and adaptive heat production.^29^ PPAR‐α improves insulin sensitivity and β‐cell function by reducing obesity, hepatic steatosis, plasma‐free fatty acids and triglycerides.^30^ Furthermore, recent studies have shown that PPAR‐α deficiency will reduce the average area of pancreatic β‐cells and reduce insulin secretion caused by glucose. Consistent with previous studies, in our study, we found that PPAR‐α produced a missense mutation in the exonic region of O‐T2DM patients, and it also showed a significant down‐regulation in mRNA expression levels (log2fold‐change = −1.03). Therefore, our results demonstrate the role of PPAR‐α in O‐T2DM, which provides new targets and therapies for the prevention and treatment of O‐T2DM.

O‐T2DM is the result of a combination of genetic and environmental factors, and changes in genetic information often lead to the development of O‐T2DM. With the development of high‐throughput sequencing technology and analytical methods, significant breakthroughs have been made in the study of the complex mechanisms of the occurrence and development of O‐T2DM. However, traditional single omics can only explain biological problems in a limited way, and the integration of experimental data of different types of multi‐omics has gradually become an inevitable trend of O‐T2DM research. Through the dimension reduction, normalization and correlation analysis of the massive data generated by high‐throughput sequencing, it can objectively reflect the change of O‐T2DM level of each group without preference and then clarify the pathogenesis and development mechanism of O‐T2DM from all‐round, multilevel and systematic, which provides new ideas and entry points for the diagnosis and treatment of O‐T2DM. The genome is the carrier and transmitter of genetic information, and it is the main factor affecting the way of life. The transcriptome is the primary means of studying gene expression by linking the genome to the proteome. Therefore, this study based on NGS sequencing technology, combined with DNA and lncRNA, to study the genes and lncRNA associated with the pathogenesis of O‐T2DM. The relationship between genomic mutation information and transcriptome expression regulation is established. On the one hand, multi‐omics evidence is mutually validated, and the results are convincing. On the other hand, the O‐T2DM development mechanism is multidimensional and comprehensively elaborated.

In the current study, we screened 3 genes (NOD2, MAP7 and ZNF429), in O‐T2DM patients for common mutations at the DNA level and differentially expressed genes at the RNA level. Nucleotide‐binding oligomerization domain 2 (NOD2) is a gene with a caspase activation and recruitment domain.^31^ Previous studies have shown that NOD2 mediates activation of the NF‐kBT transcriptional regulator family in response to different peptidoglycan fragments^32^ and that NOD2 can contribute to host defence by promoting the production of pro‐inflammatory cytokines and antimicrobial molecules.^33^ Deletion of the NOD2 gene abolished the resistance of BALA/C mice to HFD‐induced obesity,^34^ and the same phenomenon was observed in C57BL/6 mice.^35^ In line with previous studies, in this study, we found that the NOD2 gene showed missense mutations at the rs104895427 and rs5743277 sites in the exonic region of O‐T2DM patients compared with healthy subjects, and it also showed a significant down‐regulation in mRNA expression levels (log2fold‐change = −0.986). Therefore, we hypothesized that the NOD2 gene may provide some clues for the pathogenesis of O‐T2DM.

In addition, MAP7 and ZNF429 are also differentially expressed genes that are screened for common mutations and RNA levels at the DNA level in O‐T2DM patients. In our study, MAP7 was mutated at rs147645484 and rs181208871 in O‐T2DM patients compared to healthy subjects, and its expression was significantly up‐regulated at mRNA levels (log2foldchange = 1.11745). ZNF429 also has missense SNV at the rs139014529 site in the exon region of O‐T2DM patients. In addition, ZNF429 is up‐regulated in O‐T2DM patients at the transcriptional level (log2foldchange = 0.618233), compared with the normal population. Although there are no data to confirm that MAP7 and ZNF429 are relationship to obesity or T2DM, we believe that they may be closely related to the pathogenesis of O‐T2DM due to their mutations in the exon region and their significant changes in transcriptome expression levels.

In conclusion, this study analyses O‐T2DM patients from the level of exome group and screens the common mutations in O‐T2DM patients, which provided a reference for future research on the pathogenesis of O‐T2DM. The combination of exon and lncRNA was performed by high‐throughput sequencing technology, and NOD2, ZNF429 and MAP7 were screened out, which were targets at the genomic level and differentially expressed at the transcriptional level. Our study links genomic mutation information to transcriptome expression regulation and provides a molecular target for clarifying the mechanism of O‐T2DM development.

## CONFLICT OF INTEREST

The authors have no conflict of interest to declare.

## AUTHOR CONTRIBUTIONS

SHG and GJJ designed the experiments. TA and JZ wrote the manuscript. TA, JJZ, YYH, YXW, JL and TYW performed the experiments. TA, YFL, JPHH, DDZ and FFM analysed the data. All authors reviewed the manuscript.

## Data Availability

The data that support the findings of this study are available from the corresponding author upon reasonable request.
