# Supplementary material for: Combined analysis of whole‐exon sequencing and lncRNA sequencing in type 2 diabetes mellitus patients with obesity
Source: J Cell Mol Med. 2020 Jan 19;24(4):2451–63. doi: 10.1111/jcmm.14932 (PMC7028848; doi:10.1111/jcmm.14932)
Supplement: Supplementary file 1 [file JCMM-24-2451-s001.docx]

**Supplemental Table 1.** WES data quality list.

| Sample name | Raw reads | Raw data(G) | Raw depth(x) | Effective (%) | Error (%) | Q30 (%) | GC (%) |
| --- | --- | --- | --- | --- | --- | --- | --- |
| WSR001 | 42440380 | 12.73 | 210.56 | 98.06 | 0.01 | 90.22 | 51.35 |
| WSR002 | 64893202 | 19.47 | 322.05 | 98.59 | 0.01 | 91.0 | 51.95 |
| WSR003 | 39237911 | 11.77 | 194.68 | 98.37 | 0.01 | 90.52 | 51.39 |
| WSR004 | 43207150 | 12.96 | 214.37 | 97.46 | 0.01 | 91.33 | 51.63 |
| WSR005 | 46347399 | 13.9 | 229.92 | 98.06 | 0.01 | 90.87 | 51.58 |
| WSR006 | 42185602 | 12.66 | 209.41 | 95.76 | 0.01 | 92.04 | 51.67 |
| WZC001 | 43677304 | 13.1 | 216.68 | 97.33 | 0.01 | 91.01 | 51.21 |
| WZC002 | 42990725 | 12.9 | 213.37 | 97.88 | 0.01 | 91.55 | 51.41 |
| WZC003 | 46209464 | 13.86 | 229.25 | 98.15 | 0.02 | 87.91 | 50.28 |
| WZC004 | 40697598 | 12.21 | 201.96 | 98.06 | 0.01 | 91.31 | 51.45 |
| WZC005 | 40052592 | 12.02 | 198.82 | 98.33 | 0.01 | 90.73 | 51.24 |
| WZC006 | 35941107 | 10.78 | 178.31 | 98.21 | 0.01 | 91.3 | 51.83 |

**Supplemental Table 2.** Comparison rate and coverage statistics of WES data.

| Sample | Total | Mapped | Average sequencing depth | Fraction of target covered with at least 10x |
| --- | --- | --- | --- | --- |
| WSR001 | 83236262 (100%) | 83013382 (99.73%) | 131.6 | 99.50% |
| WSR002 | 127958240 (100%) | 127733434 (99.82%) | 206.11 | 99.70% |
| WSR003 | 77193986 (100%) | 76990854 (99.74%) | 125.24 | 99.50% |
| WSR004 | 84216926 (100%) | 84054519 (99.81%) | 137.88 | 99.30% |
| WSR005 | 90894820 (100%) | 90712394 (99.80%) | 148.67 | 99.60% |
| WSR006 | 80793206 (100%) | 80672839 (99.85%) | 136.8 | 99.50% |
| WZC001 | 85025244 (100%) | 84869685 (99.82%) | 131.15 | 99.30% |
| WZC002 | 84161102 (100%) | 84025587 (99.84%) | 138.32 | 99.40% |
| WZC003 | 90705668 (100%) | 90287209 (99.54%) | 130.86 | 99.30% |
| WZC004 | 79818494 (100%) | 79662505 (99.80%) | 129.49 | 99.30% |
| WZC005 | 78769464 (100%) | 78624418 (99.82%) | 127.82 | 99.30% |
| WZC006 | 70597548 (100%) | 70486046 (99.84%) | 116.3 | 99.20% |

**3.3 Variation detection results**

Based on the mapped results, we used SAMtools to identify single nucleotide variant (SNV) sites and filter SNV sites. A total of 277377 SNV sites was found in exonic. Among these SNV sites, 100 SNV sites belong to the stoploss type, which means that the substitution codon of the base becomes a non-stop codon due to substitution by one base. Subsequently, we used ANNOVAR software to annotate the SNP, which covers the location information, type, and conservative prediction of the mutation. Insertion and deletion (InDel) occurring at the coding region or splice site may alter the translation of the protein. Therefore, we separately counted the number of different types of InDel on the genome and coding region (**Supplemental Table 3 and 4**).

**Supplemental Table 3. Number of InDels on different regions of the genome**

| Sample | exonic | intronic | UTR3 | UTR5 | intergenic | ncRNA  exonic | ncRNA  intronic | splicing | ncRNA  splicing |
| --- | --- | --- | --- | --- | --- | --- | --- | --- | --- |
| WSR001 | 668 | 13231 | 620 | 436 | 7241 | 282 | 1196 | 478 | 11 |
| WSR002 | 672 | 17440 | 794 | 519 | 10705 | 323 | 1632 | 500 | 11 |
| WSR003 | 658 | 12886 | 605 | 442 | 6456 | 278 | 1145 | 452 | 9 |
| WSR004 | 660 | 12753 | 605 | 431 | 6617 | 295 | 1151 | 474 | 11 |
| WSR005 | 657 | 13603 | 653 | 473 | 7018 | 263 | 1185 | 460 | 8 |
| WSR006 | 648 | 11755 | 548 | 422 | 6162 | 273 | 976 | 465 | 16 |
| WZC001 | 658 | 12945 | 621 | 438 | 6909 | 270 | 1155 | 421 | 8 |
| WZC002 | 634 | 13180 | 632 | 458 | 6453 | 277 | 1122 | 465 | 11 |
| WZC003 | 675 | 14781 | 691 | 483 | 8878 | 320 | 1400 | 479 | 17 |
| WZC004 | 657 | 12803 | 633 | 462 | 6476 | 293 | 1126 | 447 | 14 |
| WZC005 | 639 | 12993 | 630 | 453 | 6098 | 274 | 1114 | 454 | 12 |
| WZC006 | 662 | 11517 | 546 | 425 | 4834 | 263 | 895 | 456 | 14 |

**Supplemental Table 4. Number of different types of InDel on the coding area**

| Sample | frameshift deletion | frameshift  insertion | nonframeshift  deletion | nonframeshift  insertion | stoploss | stopgain | unknown |
| --- | --- | --- | --- | --- | --- | --- | --- |
| WSR001 | 77 | 56 | 235 | 198 | 1 | 7 | 94 |
| WSR002 | 76 | 61 | 217 | 218 | 0 | 4 | 96 |
| WSR003 | 72 | 67 | 208 | 217 | 0 | 6 | 88 |
| WSR004 | 79 | 59 | 208 | 213 | 2 | 7 | 92 |
| WSR005 | 72 | 70 | 215 | 204 | 1 | 4 | 91 |
| WSR006 | 70 | 62 | 216 | 197 | 1 | 3 | 99 |
| WZC001 | 64 | 68 | 239 | 184 | 2 | 6 | 95 |
| WZC002 | 72 | 60 | 193 | 206 | 0 | 5 | 98 |
| WZC003 | 81 | 69 | 222 | 201 | 1 | 7 | 94 |
| WZC004 | 58 | 63 | 219 | 223 | 2 | 5 | 87 |
| WZC005 | 75 | 55 | 199 | 199 | 1 | 10 | 100 |
| WZC006 | 70 | 62 | 210 | 218 | 2 | 6 | 94 |

**3.4 Screening of mutation sites and classification of their harmfulness**

In addition, we performed mutation site screening on the SNP/InDel information detected by the basic analysis, and finally obtained 5607 mutation sites. Based on the priority level of the disease, here we list the top ten mutation sites (Table 2). We refer to ACMG's evidence to classify the harmfulness of the mutation sites. The number of mutation sites for each of the harmful categories obtained from the bioinformatics analysis is shown in Table 3. Finally, we performed a structural variation hazard analysis and a total of 164 sites were found (Supplemental Table 5).
